# Metabolic Changes in the Visual Cortex Are Linked to Retinal Nerve Fiber Layer Thinning in Multiple Sclerosis

**DOI:** 10.1371/journal.pone.0018019

**Published:** 2011-04-06

**Authors:** Caspar F. Pfueller, Alexander U. Brandt, Florian Schubert, Markus Bock, Bernadeta Walaszek, Helmar Waiczies, Thomas Schwenteck, Jan Dörr, Judith Bellmann-Strobl, Christian Mohr, Nicholetta Weinges-Evers, Bernd Ittermann, Jens T. Wuerfel, Friedemann Paul

**Affiliations:** 1 NeuroCure Clinical Research Center, Charité Universitaetsmedizin Berlin, Berlin, Germany; 2 gfnmediber GmbH, Berlin, Germany; 3 Institute of Neuroradiology, University Luebeck, Luebeck, Germany; 4 Physikalisch-Technische Bundesanstalt (PTB), Braunschweig und Berlin, Germany; 5 Experimental and Clinical Research Center, Charité Universitaetsmedizin Berlin and Max-Delbrueck-Center for Molecular Medicine, Berlin, Germany; Julius-Maximilians-Universität Würzburg, Germany

## Abstract

**Objective:**

To investigate the damage to the retinal nerve fiber layer as part of the anterior visual pathway as well as an impairment of the neuronal and axonal integrity in the visual cortex as part of the posterior visual pathway with complementary neuroimaging techniques, and to correlate our results to patients' clinical symptoms concerning the visual pathway.

**Design, Subjects and Methods:**

Survey of 86 patients with relapsing-remitting multiple sclerosis that were subjected to retinal nerve fiber layer thickness (RNFLT) measurement by optical coherence tomography, to a routine MRI scan including the calculation of the brain parenchymal fraction (BPF), and to magnetic resonance spectroscopy at 3 tesla, quantifying N-acetyl aspartate (NAA) concentrations in the visual cortex and normal-appearing white matter.

**Results:**

RNFLT correlated significantly with BPF and visual cortex NAA, but not with normal-appearing white matter NAA. This was connected with the patients' history of a previous optic neuritis. In a combined model, both BPF and visual cortex NAA were independently associated with RNFLT.

**Conclusions:**

Our data suggest the existence of functional pathway-specific damage patterns exceeding global neurodegeneration. They suggest a strong interrelationship between damage to the anterior and the posterior visual pathway.

## Introduction

Already in the 19^th^ century, Charcot reported a regular occurrence of neuronal and axonal degeneration beyond demyelination in multiple sclerosis (MS) [1]. Unfortunately, these findings were neglected by the research community for a long time, and consequently MS was seen as a primarily demyelinating condition, with relative preservation of axons and neurons [2]. However, within the past two decades, Charcot's initial descriptions enjoyed a revival, mainly by the advent of advanced microscopic imaging techniques, such as the combination of fluorescent immunocytochemistry with confocal microscopy. Several groups could show that independently of the demyelination process neuronal and axonal breakdown contribute to central nervous system (CNS) tissue damage and the resulting functional deficits in different stages in the course of MS [3]. It is now well accepted that MS is not only a demyelinating CNS disease but has also a considerable neurodegenerative component [4]. In the light of these findings, therapeutic strategies that specifically address the neurodegenerative component of MS are in the focus of the research. Also in neuroimaging, there is a shift of research interest from a mere depiction of the inflammatory aspects of the disease such as T2- and contrast enhancing lesion load which only correlate modestly with the clinical disease course and neurological disability (the so-called clinico-radiological paradox[5]) to improved techniques to quantify and monitor neurodegeneration. Brain atrophy is considered to represent at least partially axonal and neuronal loss in MS[6] and shows a strong association with some clinical disease-related measures. It can be quantified by various techniques, e.g. the calculation of the so-called brain parenchymal fraction (BPF) [7]–[8] but its appropriateness as primary endpoint in clinical trials on neuroprotective therapies still remains to be proven.

In recent years, optical coherence tomography (OCT) evolved as a valuable non-invasive diagnostic tool to image unmyelinated retinal CNS axons and thus to depict MS-related neurodegeneration (reviewed in [9],[10]). Based on the concept that ongoing diffuse neurodegeneration in the brain will also affect the retinal CNS axons, different groups reported reduced retinal nerve fiber layer thickness (RNFLT) in MS patients versus healthy controls[11]–[12] and could show that RNFLT correlates well with brain atrophy and physical and cognitive disability[13]–[16].

Proton magnetic resonance spectroscopy (^1^H-MRS) emerged as technique to quantify MS-related neuronal and axonal damage by measuring the brain N-acetyl-aspartate (NAA) concentration, a presumed marker of axonal and neuronal integrity (reviewed in [17]). In line with the change of paradigm on MS pathology, ^1^H-MRS provides evidence for metabolic alterations in normal appearing white matter in MS [18]–[19].

Against the background of these findings, we were interested whether changes in RNFLT indicating alterations of the anterior visual pathway are linked to impaired neuronal and axonal integrity in the visual cortex as part of the posterior visual pathway. We performed a cross-sectional study to investigate the association of RNFLT with NAA of the normal appearing white matter and the visual cortex as measured by ^1^H-MRS, and with BPF as a parameter of global brain tissue loss.

## Methods

### Participants

Using an exploratory cross-sectional study design, relapsing-remitting multiple sclerosis (RRMS) patients fulfilling the current panel criteria[20] were prospectively recruited between September 2007 and February 2009. The study was approved by the ethics committee of Charité Universitätsmedizin Berlin, Germany and all participants gave informed written consent according to the 1964 Declaration of Helsinki. Patients with MS met the following criteria: age 18-55 years, stable immunomodulatory therapy with glatiramer acetate for at least six months prior to inclusion, EDSS between 0 and 6.5, no acute relapse and no systemic steroid treatment within 30 days prior to enrolment. Patients with ophthalmologic disorders or medical conditions with impact on retinal nerve fiber layer (e.g. diabetes, glaucoma) were not included. The patients included in this study are the sub-group of patients recruited for an ongoing clinical drug trial with glatiramer acetate as required co-medication, from whom baseline data of both 1.5T MR imaging and 3T magnetic resonance spectroscopy were available. Demographic data are summarized in Table 1.

**Table 1:** Summary of demographic data, mean RNFLT, mean BPF, mean normal-appearing white matter (NAWM) NAA concentrations, mean visual cortex (VC) NAA concentrations.

	All Patients	NON/NON Patients	NON/ON Patients	ON/ON Patients
Patients (%)	86 (100)	53 (61.6)	20 (23.3)	13 (15.1)
Age, mean (range), y	41 (21–60)	40 (21–60)	41 (24–60)	41 (32–56)
Disease Duration, mean (range), m	71 (4–271)	65 (4–271)	92 (11–193)	68 (7–147)
EDSS, median (range)	2.0 (0.0–6.0)	2.0 (0.0–6.0)	2.0 (1.0–4.5)	2.5 (0–4.5)
Min. RNFLT Average, mean (SD; range), µm	91.3 (15; 46–123)	97.3 (10; 74–123)	84.6 (17.3;46–111)	75 (14.2; 56–104)
BPF, mean (SD; range)	0.851 (0.031; 0.77–0.918)	0.855 (0.032; 0.77–0.918)	0.849 (0.03; 0.791–0.913)	0.838 (0.026; 0.789–0.872)
NAWM NAA, mean (SD; range), mmol/l	13.079 (1.354; 7.652–15.807)	13.192 (1.25; 10.909–15.807)	12.994 (1.714; 7.652–15.65)	12.746 (1.212; 11.026–15.344)
VC NAA, mean (SD; range), mmol/l	13.471 (1.017; 11.176–16.086)	13.601 (1.023; 11.176–16.086)	13.43 (0.996; 11.57–14.783)	13.002 (0.948; 11.401–14.651)

(NON/NON – no previous optic neuritis, NON/ON – previous unilateral optic neuritis, ON/ON - previous bilateral optic neuritis).

### Clinical and visual assessment

Medical history, particularly with respect to visual symptoms, was taken from all study participants. Based on the documented previous history of optic neuritis (ON), we defined three subgroups of patients – patients with no, unilateral and bilateral optic neuritis. None of the study subjects had suffered from acute optic neuritis within the last 6 months before recruitment to the study. All participants underwent a complete ophthalmologic examination, including non-contact tonometry, visual acuity testing by using Snellen charts, Nieden charts and functional acuity contrast testing, spheric refractive error testing and cylindric refractive error testing. Patients who showed a non-MS related eye pathology were excluded from OCT measurements. Neurological disability in MS patients was assessed by the expanded disability status scale (EDSS) [21].

### Optical coherence tomography

RNFLT was measured with a Stratus 3000 OCT (Carl Zeiss Meditec, Dublin, California) using the “fast RNFL 3.4” protocol (software version 4.0). Three 3.4 mm diameter circular scans were acquired over 1.92 seconds. A good quality image was defined as an image with generalised signal distribution, a reflectance signal from either RNFL or retinal pigment epithelium strong enough to identify either layer, no missing parts caused by eye movements, and a signal strength of ≥8 of 10 [22]. The segmentation line defining the upper and lower border of the RNFL was required to be on the internal limiting membrane and lower border of the RNFL. Images which did not meet these criteria were excluded. The OCT A-scan data were digitally exported in a blinded fashion.

### Magnetic resonance imaging and brain parenchymal fraction calculation

MRI measurements were performed on a 1.5 tesla scanner (Avanto, Siemens Medical Systems, Erlangen, Germany). A three-dimensional T1-weighted image (MPRAGE) was acquired according to the following protocol: T_R_ 1.9 ms, T_E_ 3.09 ms, T_I_ 1.1 ms, flip angle 15°, matrix size 1 mm^3^. Brain tissue volume, normalized for subject head size, was estimated applying SIENAX[23]–[24],part of FSL [25]. SIENAX starts by extracting brain and skull images from the single whole-head input data [26]. The brain image is then affine-registered to MNI152 space (using the skull image to determine the registration scaling) [27]–[28] in order to obtain the volumetric scaling factor to be used as normalization for head size. Next, tissue-type segmentation with partial volume estimation is carried out in order to calculate total volume of brain tissue [29].

### MR spectroscopy

**Figure 1:**
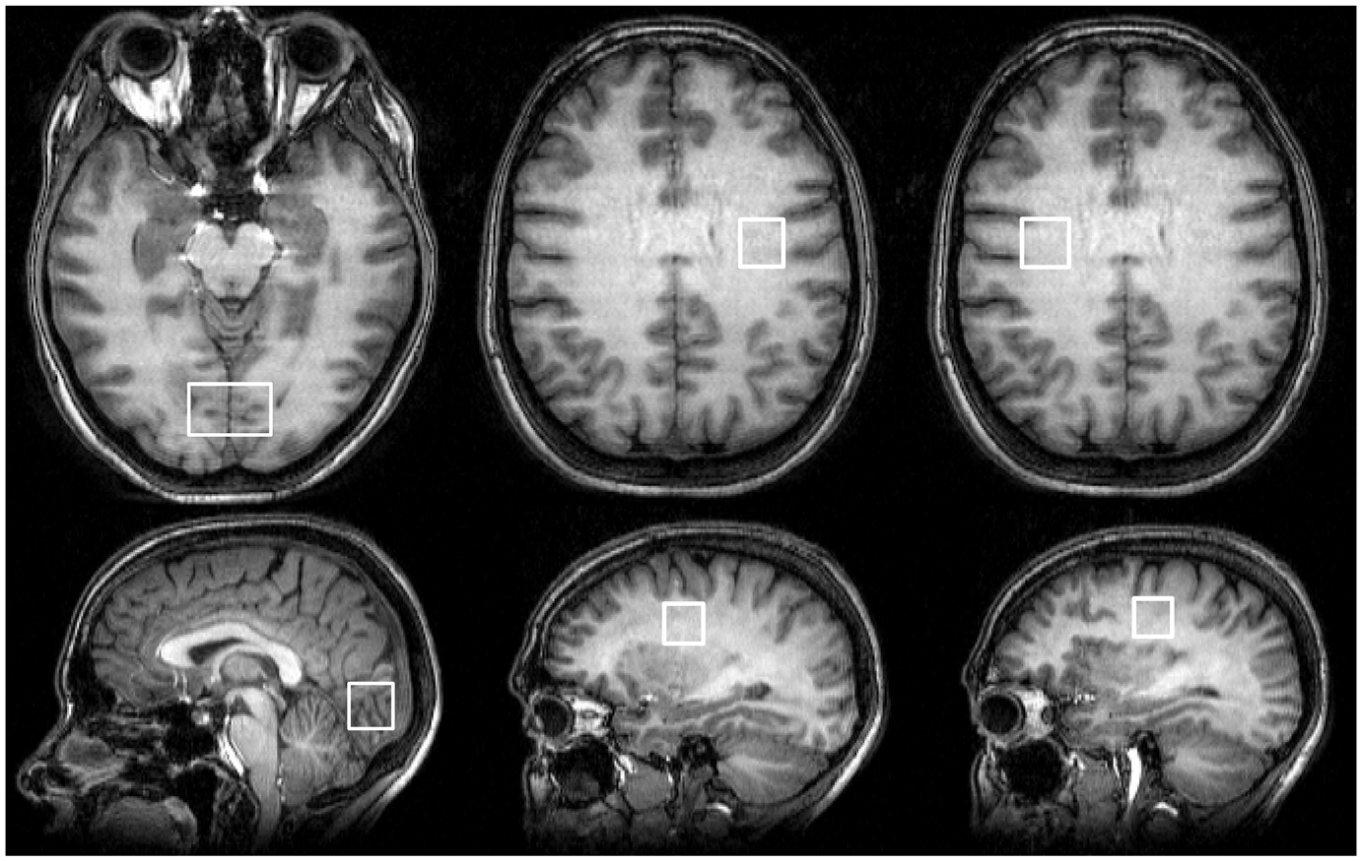
^1^H-MRS voxel placement. Visual representation of typical voxel placement for MR spectroscopy. In each patient, NAA concentrations were measured in a visual cortex voxel (VC) and two normal-appearing white matter voxels (NAWM).

MR measurements were carried out on a 3 tesla scanner (MEDSPEC 30/100, Bruker Biospin, Ettlingen, Germany). T_1_-weighted images were acquired using MDEFT (modified driven equilibrium Fourier transform, with T_E_ = 3.8 ms, T_R_ = 20.53 ms; 128 contiguous slices, 1.5 mm thick; 1-mm in-plane (x–y) resolution). After localized shimming, magnetic resonance spectra were recorded from two voxels located in left and right normal appearing periventricular white matter (2×2×2 cm^3^), and a voxel centered on the visual cortex (3×2×2 cm^3^) (Fig. 1). The PRESS (point resolved spectroscopy) sequence preceded by water suppression (3 Gauss CHESS pulses of 25.6 ms duration) was used throughout. Details of the procedure for metabolite quantification were previously published [30]. For one metabolite spectrum eight subspectra of 16 phase cycled scans each were recorded with T_R_ = 3 s and T_E_ = 80 ms. Before further processing, the 8 metabolite subspectra were corrected for eddy currents using water-unsuppressed spectra (T_R_ and T_E_ as above), automatically corrected for frequency and phase shifts, and added together to give 128 averages. Spectral quantification was carried out using a time domain-frequency domain fitting procedure that involves background estimation by regularization [31]. Any residual contributions by macromolecules are accommodated in the baseline by the fitting procedure. Mean uncertainties corresponding to Cramér-Rao lower bounds with added uncertainties from the background modelling[31] for the fitting of NAA were as small as 2.1% for the visual cortex voxel and 2.4% for the normal-appearing white matter voxels. The fitted NAA amplitudes were corrected for different coil loading by an aqueous metabolite phantom used for spectrum analysis and the individual subject's head (principle of reciprocity), and for transverse relaxation effects using mean T_2_ values measured earlier at 3 T for normal-appearing white matter [32] and cortical regions [30]. Longitudinal relaxation effects were neglected because T_1_ was assumed to be similar in the aqueous phantom and in brain tissue. Metabolite concentrations were corrected for cerebrospinal fluid (CSF) in the voxels studied by using the CSF fractions obtained by segmenting the T_1_-weighted images with SPM2 (www.fil.ion.ucl.ac.uk/spm/spm2.html).

### Statistical analysis

RNFLT, ^1^H-MRS and BPF data were analyzed for normal distribution using skewness and kurtosis of data histograms. All data was within +/-1.5 skewness and kurtosis. Additionally, Shapiro-Wilk tests were performed to check for normal distribution. According to these tests, RNFLT, BPF, visual cortex NAA were normally distributed whereas NAA in normal-appearing white matter was not (Shapiro-Wilk test, p = 0.037).

Correlation between normal-appearing white matter NAA and visual cortex NAA and BPF was assessed using Pearson's correlation coefficient, counterchecked with Spearman's correlation due to the distribution of the normal-appearing white matter NAA. Association of normal-appearing white matter and visual cortex NAA and BPF with RNFLT was tested with Generalized Estimating Equation Models (GEE) to adjust for inter-eye dependencies within patients using RNFLT as the dependent variable and BPF, visual cortex voxel NAA or normal-appearing white matter NAA as single independent variables. Finally, a GEE with BPF and visual cortex NAA as independents and RNFLT as dependent variable was used to calculate the independent association of BPF and visual cortex NAA with RNFLT (combined model). Since GEE function in PASW 18 does not provide standardized output for coefficients, we approached this issue in the following way: RNFLT, BPF and visual cortex NAA were transformed to standardized z-values and each GEE was performed again with these z-values instead of the original values (standardized Beta, see Table 2).

**Table 2:** Statistical data for GEE and combined model GEE (NAWM = normal-appearing white matter, VC = visual cortex).

	Variable	Dependent Variable	B (Std. Error; 95% CI)	standardized B (Std. Error; 95% CI)	Chi-Square	*P* value
GEE 1	VC-NAA	RNFLT	2.823 (1.4238; .033–5.614)	.191 (.0964; .002–.380)	3.932	.047
GEE 2	BPF	RNFLT	132.907 (39.941; 54.625–211.190)	.269 (.0809; .111–.428)	11.073	.001
GEE Combined model	BPF	RNFLT	120.448 (38.3810; 45.223–195.673)	.244 (.0777; .092–.396)	9.848	.002
	VC-NAA	RNFLT	2.784 (1.3720; .095–5.473)	.188 (.0929;.006–.370)	4.117	.042

Analyses of variance were performed with BPF, visual cortex NAA or normal-appearing white matter NAA as dependent variable and history of ON as nominal independent factor to identify group differences regarding BPF, visual cortex NAA and normal-appearing white matter NAA between patients with history of bilateral optic neuritis, patients with history of unilateral optic neuritis and without previous optic neuritis. Differences in age, EDSS and disease duration between these groups defined by history of optic neuritis were assessed with Kruskal-Wallis tests, differences in gender with Pearson's Chi Square analysis.

All statistical tests were performed using PASW 18 (SPSS, Chicago, IL, USA). For all calculations, statistical significance was established at p<0.05. Data sets with partly missing data as indicated under results were not excluded from sub-analyses. All tests should be understood as constituting exploratory data analysis, such that no adjustments for multiple testing were made.

## Results

86 RRMS patients were recruited. Three patients were excluded from OCT analysis due to non-MS related retinal pathologies. All other eyes were included and were analyzable with an RNFLT signal strength ≥8. Data from ^1^H-MRS measurements were available for all patients. In five patients BPF analysis was not performed due to insufficient image quality (e.g. motion artifacts). Patients in the three subgroups defined by history of optic neuritis did not differ significantly regarding age (Kruskal-Wallis, p = 0.947), disease duration (Kruskal-Wallis, p = 0.172), EDSS (Kruskal-Wallis, p = 0.829) or gender (Chi-Square, p = 0.768). Clinical and demographical data including history of optic neuritis, RNFLT, BPF and ^1^H-MRS are given in Table 1.

### BPF correlates with RNFLT but not with NAA concentration in visual cortex and normal-appearing white matter

**Figure 2:**
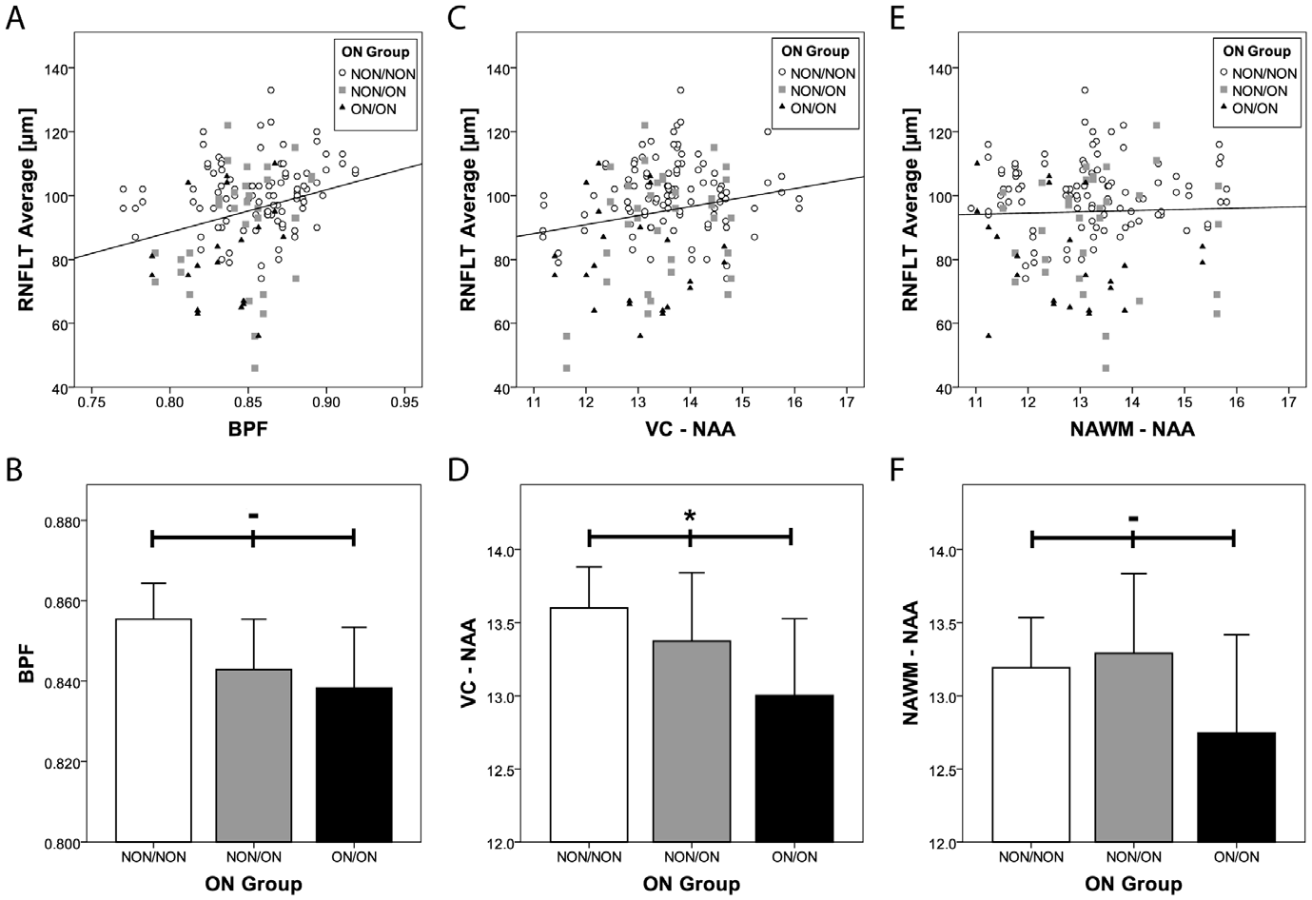
Correlation of RNFLT with BPF and ^1^H-MRS parameters. a) Depicted is the average RNFLT, every symbol representing a single eye examined together with the corresponding BPF values. The symbols represent the patient's previous history of optic neuritis (open circles – no previous optic neuritis, grey squares - unilateral optic neuritis, black triangles – bilateral optic neuritis) A linear correlation function was calculated by a Generalised Linear Model to account for intra-individual inter-eye relationships (p = 0.001). b) Mean BPF was calculated for three groups that were defined based on their previous history of optic neuritis (white bar– no previous optic neuritis, grey bar - unilateral optic neuritis, black bar – bilateral optic neuritis). The (-) symbol indicates a trend, but a missing significant correlation of group differences as calculated by ANOVA (p = 0.055). Error bars represent 2× standard error of the mean (SEM). c) RNFLT averages are shown in relation to corresponding NAA concentrations in the visual cortex (VC). The symbols are coded as in a). The correlation is significant (p = 0.047). d) Mean visual cortex voxel (VC) NAA and the significance of group differences was calculated for optic neuritis groups as in b). The asterisk indicates statistically significant (p = 0.046) group differences. Error bars represent 2× standard error of the mean (SEM). e) RNFLT averages are shown in relation to corresponding NAA concentrations in normal-appearing white matter (NAWM). The symbols are coded as in a). No significant correlation was found (p = 0.531). f) Mean NAA in normal-appearing white matter (NAWM) and the significance of group differences was calculated for optic neuritis groups as in b) (p = 0.429). Error bars represent 2× standard error of the mean (SEM).

BPF correlated with RNFLT (GEE, CI95% low  = 0.55 µm/%, high  = 2.11 µm/%, p<0.001). There was no correlation between visual cortex NAA concentration and BPF (Pearson, p = 0.161) or normal-appearing white matter NAA concentration and BPF (Pearson, p = 0.540). Comparing the BPF in the three subgroups defined by history of optic neuritis there was a trend towards lower BPF in patients with previous episodes of optic neuritis (ANOVA, p = 0.055) (Figure 2A and B). Further statistical details, including a calculated standardized coefficient, are provided in Table 2.

### RNFLT correlates with NAA concentration in the visual cortex but not in the normal-appearing white matter

We found a correlation between RNFLT and visual cortex NAA concentration (GEE, Confidence Interval (CI95%) low  = 0.03 µm/(mmol/l), high  = 5.61 µm/(mmol/l), p = 0.047). The subgroups regarding history of optic neuritis differed in their visual cortex NAA concentration, indicating that patients with previous unilateral or bilateral optic neuritis exhibited lower NAA levels than those without optic neuritis (ANOVA, p = 0.046), (Figure 2C and D). There was no correlation between RNFLT and NAA concentration in the normal-appearing white matter (GEE, CI95% low  =  −1.26 µm/(mmol/l), high  = 2.44 µm/(mmol/l), p = 0.531), nor a difference in normal-appearing white matter NAA concentration between subgroups regarding the history of optic neuritis (ANOVA, p = 0.429) (Figure 2E and F). (Further statistical details, including a calculated standardized coefficient, are provided in Table 2.)

### BPF and visual cortex NAA concentrations are independently associated with average RNFLT

Using visual cortex NAA concentration and BPF as independent variables in a multivariate GEE analysis, we found that both BPF (CI 95% low  = 0.45 µm/%, high  = 1.96 µm/%, p = 0.002) and visual cortex NAA concentration (CI 95% low  = 0.10 µm/(mmol/l), high  = 5.47 µm/(mmol/l), p = 0.042) are independently associated with RNFLT. Further statistical details, including a calculated standardized coefficient, are provided in Table 2.

## Discussion

This cross-sectional study is the first to investigate MS-related axonal and neuronal damage in a large number of patients by three different imaging modalities including OCT, brain atrophy measurement by MRI, and ^1^H-MRS of the visual cortex and normal-appearing white matter at 3 T. Our main findings are that (i) RNFLT is correlated with NAA concentration in the visual cortex but not in the normal-appearing white matter, (ii) visual cortex NAA concentrations are lower in patients with previous optic neuritis than in those without, (iii) both visual cortex NAA and BPF are independently associated with RNFLT, and (iv) BPF and RNFLT show a significant association.

The novel multimodal imaging approach merging OCT and MRI and the additional application of ^1^H-MRS at 3 T, yielding an improved signal-to-noise ratio compared to 1.5 T[17] enabled us to investigate not only the relationship between brain atrophy and RNFL reduction which had already been assessed previously in smaller patient cohorts[13] [15] and by us in a larger patient cohort (Dörr et al., submitted), where we analyzed the association of RNFLT and the total macular volume with global brain atrophy, but to evaluate also the association between disease-related damage of the anterior part (RNFLT by OCT) and that of the retrogeniculate part of the visual pathway (NAA in the visual cortex by ^1^H-MRS). Thus, our combined OCT and ^1^H-MRS data may suggest an interconnection of MS-associated neurodegeneration in both parts of the visual pathway. The multivariate statistical model revealed that the correlation of RNFLT reduction with lower NAA concentrations is not a mere consequence of global brain tissue loss as one could assume given the correlation of RNFLT with BPF in both our work and that of others [13] [15]. On the contrary, loss of NAA in the visual cortex appears to be associated with thinning of the RNFL independently from brain atrophy. These findings could indicate that – beyond an undoubted diffuse neurodegenerative process in MS which is detectable by measurement of global brain tissue loss and also by RNFL measurements - additional progressive neurodegenerative damage may evolve in specific tracts or functional systems such as the visual pathway. This assumption is further supported by previous studies describing visual pathway damage in MS by means of voxel-based morphometry, diffusion tensor imaging or magnetization transfer ratio[33]–[35], and damage to other functional systems such as pathways involved in learning and memory [36]–[37]. The connection between the anterior and posterior visual pathway damage raises the question of a mutual interdependency of these alterations and implicates the possible existence of transsynaptic damage processes in the anatomical correlates of the visual pathway in MS. This concept has already been described recently for glaucoma[38]–[39] and in the context of amblyopia[40] and in congenital or acquired homonymous hemianopia [41]. In this regard, the lateral geniculate nucleus (LGN) as region of change-over from axons deriving from the anterior visual pathway to neurons from which axons forming the optic radiation emerge is of importance. Interestingly, a histopathological study of neuronal changes in the LGN by Evangelou et al. [42] strongly supports the concept of transsynaptic degeneration. In this context, Green et al. could show just recently by a larger histopathological study, that retinal atrophy and intraretinal inflammation may exceed previous assumptions, indicating that also other structures of the foremost part of the visual pathway, as the retinal inner nuclear layer, may be affected by transsynaptical axonal and neuronal degeneration, not only the retinal nerve fiber layer [43]. This hypothesis is further supported by our findings of lower RNFL thickness in patients with previous optic neuritis compared to those without, in line with earlier reports[12] [44]–[45], and concordantly also lower visual cortex NAA concentrations in patients with previous optic neuritis. However, the underlying pathophysiological mechanisms remain to be elucidated, including the direction of damage cascades (anterograde, retrograde) and their temporal evolution. These mechanisms cannot be deduced from cross-sectional studies, as these only provide a description of the current status within a narrow time-frame.

A methodological limitation of our study is the lack of an additional MRS voxel containing mixed or gray matter in another brain region than the visual cortex. Preferably, a separate cortex voxel representative for an independent functional pathway, such as a voxel covering the motor cortex, could have been used as a more appropriate control region, which could not be applied in our study to limit scan time to an acceptable extent. The reduced cortical NAA concentrations and RNFL thinning may not be specific for the visual cortex but might also have been detected in other cortex regions, thus only indicating a relationship between two different parameters depicting global neurodegeneration. However, despite this limitation we believe that our findings from this exploratory study could support the hypothesis of tract specific damage to the visual pathway in MS as (i) NAA only in the visual cortex voxel comprising both gray and white matter but not NAA in the normal-appearing white matter voxel correlated with RNFLT, and (ii) our subgroup analysis showed that the extent of NAA reduction in the visual cortex voxel is related to the history of optic neuritis. Notwithstanding, future studies on visual pathway damage in MS should include additional cortical MRS voxel if possible. In addition, the advent of novel spectral-domain OCT devices with an improved spatial resolution and a better retest-reliability, that replace the current time-domain OCT devices in the future, will also contribute to a more accurate description of the pathology of visual pathway damage on a morphological level [46]–[47].
